# Analyse de la rétention dans les soins des personnes vivant avec le VIH au Togo : résultats d'une enquête menée en 2021

**DOI:** 10.48327/mtsi.v5i1.2025.664

**Published:** 2025-03-13

**Authors:** Abla Sefako AKAKPO, Julienne Noude TECLESSOU, Kodjo DEKU, Jean-Paul TCHUPO, Souley WADE, Didier Koumavi EKOUEVI, Zakilatou ADAM, Anoumou Yawotsè DAGNRA, Palokinam PITCHÉ

**Affiliations:** 1 Service de dermato-vénéréologie, Université de Lomé, Togo; 2 Conseil national de lutte contre le sida et les IST, Togo; 3 Family health International 360; 4 Département de santé publique, Université de Lomé, Togo; 5 Programme national de lutte contre le sida, les hépatites et les IST, Togo; 6 Laboratoire de biologie moléculaire, Université de Lomé, Togo Autrice

**Keywords:** PVVIH, Antirétroviraux, Rétention dans les soins, Perdus de vue, Soins de santé, Togo, Afrique subsaharienne, PLWH, Antiretrovirals, Retention in care, Lost to follow-up, Health care, Togo, Sub-Saharan Africa

## Abstract

**Introduction:**

Le but de notre étude était d'analyser la rétention dans les soins des personnes vivant avec le VIH (PVVIH) sous antirétroviraux (ARV), et leur survie à 12, 24 et 36 mois.

**Méthodes:**

Il s'agit d'une analyse transversale rétrospective d'une cohorte de PVVIH âgées de 15 ans et plus qui ont initié un traitement par ARV (TARV). Un échantillonnage raisonné a permis de prendre en compte l'activité des différents centres de soins et les contraintes budgétaires (approche quantitative). Y ont été associés des entretiens individuels approfondis et des *focus groups* (approche qualitative).

**Résultats:**

Durant la période d’étude, 2 100 patients infectés par le VIH ont été inclus. L’âge médian des patients était de 44 ans (intervalle interquartile (IIQ) [36-51]) avec une différence statistiquement significative selon le sexe (p<0,001), les femmes étant moins âgées que les hommes (42 ans *versus* 46 ans). La durée médiane sous TARV était de 5 ans (IIQ [2-8]) sans différence statistique selon le sexe (p=0,752). Au jour de l'enquête, 20,5 % (n=431) et 25,1 % (n=509) étaient perdus de vue (PDV) respectivement 90 jours et 28 jours après la date de visite programmée. On dénombrait 146 adultes décédés, soit une mortalité brute de 6,9 % avec un IC à 95 % [5,9-8,1]. Environ 60 % des 158 PVVIH retenues de façon aléatoire parmi nos patients PDV avaient pu être joints au téléphone et continuaient toujours le traitement selon leurs déclarations. La rétention dans les soins était de 72,5 % des patients et la probabilité de poursuivre le traitement de 91,6 % à 12 mois, de 87,8 % à 24 mois et de 78,7 % à 60 mois. La rétention dans les soins était plus marquée chez les femmes et plus importante chez les PVVIH âgées de 35 ans et plus lors de l'initiation du traitement dans les formations sanitaires offrant toute la gamme des activités (soins et traitement, recherche active des patients, présence de médiateurs sociaux).

**Conclusion:**

Notre étude réalisée dans la période de la pandémie de Covid-19, montre des taux acceptables de rétention dans les soins des PVVIH. Ces résultats ont permis de proposer des approches de solutions pour améliorer le programme de prise en charge dans le pays : l'harmonisation des procédures de relance des patients PDV avec la mise en place de leur recherche active (avec l'aide des médiateurs communautaires); la dispensation pour trois ou six mois des médicaments ARV aux PVVIH.

## Abstract (en)

**Introduction:**

The aim of our study was to analyze retention in care and survival at 12, 24, and 36 months among people living with HIV (PLWH) on antiretroviral therapy (ART).

**Methods:**

This is a retrospective cross-sectional analysis of a cohort of PLWH aged 15 years and older who started ART. Purposive sampling was used, taking into account the activities of different health centers and budgetary constraints (quantitative approach). In-depth individual interviews and focus groups were also conducted (qualitative approach).

**Results:**

During the study period, 2,100 HIV-infected patients were enrolled. The median age of patients was 44 years (interquartile range (IQR) [36-51]), with a statistically significant difference according to sex (p< 0.001), with women being younger than men (42 *versus* 46 years). The median duration of ART was 5 years (IQR [2-8]) with no statistical difference by gender (p=0.752). At baseline, 20.5% (n=431) and 25.1% (n=509) were lost to follow-up (LTF) 90 days and 28 days after scheduled visit, respectively. There were 146 adult deaths, for a crude mortality rate of 6.9% (95% CI [5.9-8.1]). Approximately 60% of the 158 PLWH randomly selected from our LTF patients could be reached by telephone. They reported that they were still in care. Retention in care was 72.5%, and the probability of retention was 91.6% at 12 months, 87.8% at 24 months, and 78.7% at 60 months. Retention in care was more pronounced among women and more significant among PLWH aged 35 years and older when treatment was initiated in health care facilities offering the full range of activities (care and treatment, active patient search, presence of social mediators).

**Conclusion:**

Our study, conducted during the Covid-19 pandemic, shows acceptable retention rates in care for PLWH. These results make possible to propose solutions to improve the care program in the country: the harmonization of procedures for tracing PLWH with the implementation of their active search (with the help of community mediators) and the dispensing of ART to PLWH for three or six months.

## Introduction

La région africaine de l'Organisation mondiale de la santé (OMS) concentre, à elle seule, plus de deux tiers des personnes vivant avec le VIH (PVVIH), soit environ 25,7 millions en 2019 [10]. Le traitement antirétroviral (TARV) est bénéfique pour le patient parce qu'il permet de réduire la mortalité et la morbidité [3]. Mais encore faut-il que les PVVIH ne quittent pas le circuit des soins, c'est-à-dire ne suspendent pas leur traitement ou ne soient pas perdus de vue (PDV). En devenant PDV, les patients mettent directement en danger leur propre santé mais aussi celle des autres. Selon l’OMS, la rétention dans les soins VIH est définie comme un engagement régulier d'un patient dans les soins médicaux, après initiation du TARV. Le patient n'est ni PDV, ni décédé dans l'intervalle entre deux visites [11]. La rétention dans les soins des patients sous ARV est un défi majeur dans les programmes de lutte contre le VIH.

Le nombre de patients sous TARV qui sont PDV est variable d'un pays à l'autre. D'après une étude réalisée en 2010 par Ekouevi et *al.* en Afrique de l’Ouest (Bénin, Côte d’Ivoire, Gambie, Mali, Sénégal), la probabilité de rétention dans le programme de soins était de 90 % à 3 mois, 84 % à 6 mois et 76 % à 12 mois [2]. Toutefois, les comparaisons entre les études devraient se faire avec précaution parce qu'il n'existe pas de consensus pour définir un PDV chez les PVVIH de façon générale. Chez les adultes, la proportion de PDV variait entre 13,9 % et 18,3 % en Éthiopie [7, 17] et était de 21,2 % au Nigéria [13].

Au Togo, selon les résultats de l’étude sur les indicateurs d'alerte précoce (IAP) réalisée en 2017, la rétention dans les soins des PVVIH sous TARV 12 mois après l'initiation du traitement était de 91 % [14]. Entre 2008 et 2011, Saka *et al.* avaient rapporté, sur un total de 16 617 patients, une proportion de PDV de 1,7 % avant 6 mois et de 5,6 % après 6 mois [15]. Dans le cadre du programme d'accélération mis en place dans le pays, on observe une augmentation significative du nombre de PVVIH en 2021 avec un taux de couverture thérapeutique de 75 % [14]. Afin de s'assurer de la qualité du programme de prise en charge (PEC), il était important de documenter tous les aspects dudit programme, notamment la problématique de rétention dans les soins des patients sous traitement dans les centres de santé. Par ailleurs, les deux années (2020 et 2021) de la pandémie du Covid-19 ont éprouvé les systèmes de santé en Afrique et au Togo avec un impact négatif sur les programmes de PEC des maladies chroniques comme le VIH.

Ainsi, à la sortie de cette pandémie au Togo, il était important de mener la présente étude dont le but était d'estimer d'une part, le taux de PDV des PVVIH à 28 jours et à 3 mois après la mise sous ARV et d'autre part, le taux de rétention dans les soins à 6, 12 et 24 mois des PVVIH dans les sites de PEC, ainsi que les facteurs associés à la rétention dans les soins.

## Méthodes

### Schéma, période couverte par l'étude et sélection des sites d'étude

Une approche mixte associant des méthodes quantitatives et qualitatives a été utilisée. Il s'agissait d'une analyse transversale d'une cohorte rétrospective des dossiers des PVVIH âgées de 15 ans et plus ayant initié un TARV. L’étude rétrospective a couvert une période de 13 mois, du 1/12/2021 au 28/01/2022, dans les six régions sanitaires du Togo. En 2019, on dénombrait 670 centres de PEC du VIH. Les centres de PEC ont été inclus dans l’étude au terme d'un choix raisonné prenant en compte le budget disponible pour l’étude, l'accessibilité des centres et la taille de la file active sur chaque centre. Ainsi dans chaque région sanitaire, quatre centres ont été sélectionnés en fonction de la file active, soit 2 grands centres de PEC (file active ≥ nombre médian de PVVIH dans la région soit ≥ 50) et 2 petits centres de PEC (file active < nombre médian de PVVIH dans la région soit < 50). De plus, dans chacune des trois régions sanitaires (Grand Lomé, Maritime et Plateaux) où existent des centres du projet *Ending AIDS in West Africa* (EAWA), deux de ces centres ont été retenus, s'ajoutant ainsi aux quatre précédents. Au total, 30 centres ont été inclus comptant 24 196 inscrits dans la file active (68 164 dans l'ensemble du pays) [13]. Les centres prenant en charge moins de 30 PVVIH n'ont pas été retenus.

### Population d'étude

Pour l'enquête quantitative : au total, 2 100 dossiers ont été inclus par une approche d’échantillonnage conçue pour prendre en compte les pondérations des sites et des régions et pour permettre une comparaison des sites du Programme PEPFAR *(President's Emergency Plan for AIDS Relief)* et non PEPFAR. Le PEPFAR est le Plan présidentiel d'urgence pour la lutte contre le sida (une initiative du gouvernement américain), offrant toute la gamme des activités : soins et traitement, recherche active des patients, présence de médiateurs sociaux. La répartition de l’échantillon attribué aux régions a été faite à la proportionnelle de la taille de la file active. Sur chaque site, la sélection du nombre de dossiers a été faite par tirage au sort aléatoire simple.

D'après les données de la littérature, la rétention dans les soins VIH en Afrique varie en général entre 60 et 80 %. Pour un niveau de confiance à 95 %, une rétention attendue à 70 % avec une marge d'erreur à 2 %, et en considérant 10 % de données inexploitables, il fallait inclure au minimum 1 831 patients.

Le calcul de la taille de l’échantillon a été faite sur la base de la formule :

n=[Z^2^*p (1-p)]/Δ^2^
n : taille minimale de l’échantillon à inclure,
Z : niveau de confiance (la valeur type du niveau de confiance à 95 % sera 1,96),
p : proportion attendue de la population qui présente la caractéristique étudiée,
Δ : marge d'erreur [11].

Pour l'enquête qualitative : des entretiens individuels approfondis et des *focus groups* ont été réalisés sur un échantillon de convenance issu des différents groupes de personnes : les patients PDV et retrouvés, les prestataires de soins ARV (médecins, pharmaciens, infirmiers…), les travailleurs communautaires et sociaux des sites d’étude. Au total, trois *focus group discussion* (FGD) ont été mis en place par région : un FGD avec 5 à 10 PVVIH sous ARV PDV et retrouvés; un FGD avec 5 à 10 personnes impliquées dans la prise en charge des PVVIH sous ARV; un FGD par région avec 5 à 10 travailleurs communautaires.

### Définition des concepts

- Rétention dans les soins : toute PVVIH encore suivie dans les soins sous ARV ni décédée, ni PDV (sur le plan statistique elle a été définie comme [1- (proportion des PDV + proportion de décès)].
- Rétention brute : proportion de personnes ni PDV ni décédé au moment de l'enquête.
- Probabilité d’être retenu dans les soins au temps i : probabilité pour 100 personnes rentrant dans les soins d’être encore suivies au temps i.
- Perdu de vue (PDV) : PVVIH qui n'a pas été vue au centre depuis 90 jours au moins (3 mois) à compter de la date prévue du dernier rendez-vous. Cette définition ne tient pas compte de PDV de 28 jours, car il s'agit d'un indicateur spécifique du programme EAWA et non d'une recommandation au niveau national.
- Vrais PDV : les PVVIH qui sont déclarées PDV sur leur site de PEC initial, mais qui ne sont pas suivies ailleurs.
- Attrition : cela comprend les décédés et les PDV (PDV déclarés dans le dossier et patients non revenus au centre dans les 90 jours après la date de visite programmée). La variable rétention est définie comme le complément à 1 de l'attrition (1-attrition).
- Interruption de traitement (IT) : absence de contact clinique (rendez-vous pour remise des ARV) pendant 28 jours après la date du dernier rendez-vous (RDV). L'interruption de traitement à 28 jours est un indicateur spécifique mesuré dans le cadre du projet EAWA.
- Devenir des sujets déclarés PDV après 90 jours : nous avons procédé à un suivi téléphonique sur un échantillon de 158 patients PVVIH tiré de façon aléatoire (mais proportionnellement aux PDV dans les six régions) parmi les patients déclarés PDV. La recherche de PDV est faite par les médiateurs sociaux, les agents de santé communautaires, ainsi que les réseaux existants de PVVIH. Une fois que le patient a été déclaré comme PDV par le site, cette approche utilise les téléphones mobiles et les visites à domicile pour retrouver ces patients ou s'assurer qu'ils sont encore en vie.

### Collecte des données

Elle a été réalisée du 01/12/2021 au 28/01/2022 par la revue des registres et dossiers médicaux des patients sous TARV. En outre, des médiateurs ont joint les patients PDV par téléphone et utilisé un guide d'entretien pour recueillir les informations. Des *focus groups* ont été organisés au sein des populations suivantes : les responsables des sites; les PVVIH PDV et retrouvées; les travailleurs communautaires; le personnel impliqué dans la PEC.

Pour l’étude de cohorte, une fiche d'extraction des données a été élaborée comprenant une trentaine de variables. Un codage des données cliniques a été rapporté sur la fiche d'extraction en mentionnant les données manquantes qui ont été prises en compte dans l'analyse. Les données ont ensuite été saisies dans une base de données élaborée sous le logiciel EpiData version 4.1.

Pour la recherche des PDV, une analyse des bases de données, une enquête par appel téléphonique par des médiateurs, ainsi qu'une revue des carnets des PVVIH ont été réalisées pour croiser les données et documenter les raisons de la perte de vue. Les entretiens qualitatifs ont été réalisés par une équipe de psychologues grâce à des guides d'entretien développés à cet effet.

### Analyse des données

Après la finalisation de la collecte des données, les données quantitatives ont été exportées afin de les analyser avec le logiciel R version 4.0.1. Les variables qualitatives ont été décrites sous forme d'effectif et de proportion. Les variables quantitatives ont été décrites par leur moyenne ou médiane et leurs paramètres de dispersion (écart type et quartiles). La méthode de Kaplan-Meier a été utilisée pour estimer la probabilité de décès ou de PDV ou de rétention dans les soins avec leur intervalle de confiance (IC) à 95 %.

Nous avons utilisé des modèles de régression de Cox pour identifier les facteurs associés à la rétention dans les soins. La rétention dans les soins a été codée comme une variable binaire, avec une modalité « Non » si le patient était PDV ou décédé, et une modalité « Oui » dans le cas contraire. Le modèle a inclus tous les patients pour lesquels les données étaient complètes. Les rapports de risque instantanés ont été estimés avec leur intervalle de confiance à 95 %. En analyse univariée, les variables ayant une valeur de p inférieure à 0,2 ont été incluses dans un modèle multivarié. L'analyse multivariée a été réalisée par une approche pas-à-pas descendante.

Les données qualitatives issues des enregistrements des entretiens individuels et des entretiens de groupe ont été transcrites au moyen du logiciel Word et analysées à l'aide de la méthode « d'analyse thématique » [8].

### Considérations éthiques

Le protocole d'enquête a été validé par le comité bioéthique pour la recherche en santé (CBRS) du ministère de la santé du Togo (avis n^0^ 053/2021/ CBRS du 18 novembre 2021). Un consentement éclairé écrit a été obtenu auprès de chaque personne avant son inclusion dans l’étude. Avant d'enrôler des PVVIH de moins de 18 ans dans les *focus groups*, le consentement oral des parents ou du tuteur a été obtenu, en plus de l'assentiment des adolescents. Un code d'identification a été attribué à chaque patient avant la saisie des données. L'accès à la base de données était sécurisé par un mot de passe. Tous les enquêteurs ont signé une clause de confidentialité au moment de leur recrutement. Ainsi, l'anonymat des patients et la confidentialité des données collectées ont été garanties. Les outils de collecte des données ne contenaient aucune information pouvant permettre une violation de l'anonymat des patients.

## Résultats

### Approche quantitative

#### Caractéristiques sociodémographiques

**Figure 1:**
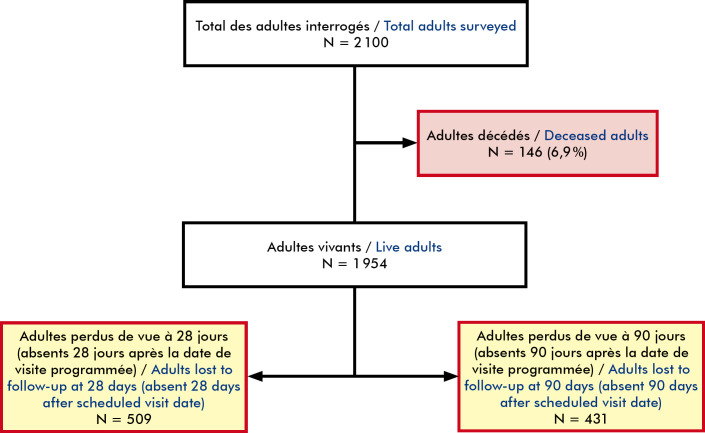
Différents indicateurs de suivi des adultes

Au total, 2 100 patients infectés par le VIH ont été inclus dans cette étude. Globalement, les indicateurs (âge, sexe, statut matrimonial, stade clinique, type d’établissement, PDV) montrent des tendances communes, mais avec des variations régionales notables (Tableau I). L’âge médian des patients était de 44 ans (IIQ [36-51]) avec une différence statistiquement significative selon le sexe (p<0,001) et entre les régions. Les moins de 50 ans représentaient 70 % des patients. Les femmes étaient moins âgées que les hommes (42 ans versus 46 ans, p < 0,001). Pour 20 % des patients inclus dans l’étude, le diagnostic remontait à plus de 10 ans. Les données de la charge virale étaient manquantes pour 96 % des patients et 31 % avaient bénéficié d'un changement de régime ARV. La durée médiane sous TARV était de 5 ans (IIQ [2-8]) sans différence statistique selon le sexe (p=0,752). Selon les estimations basées sur les définitions opérationnelles, 20,5 % des patients étaient PDV au RDV 90 jours après la date de visite programmée (n=431) et 25,1 % étaient PDV au RDV 28 jours après la date de visite programmée (n=509) (Fig. 1). On note une forte variation régionale : taux plus élevé en région Centrale (30,5 %) et plus faible dans les Savanes (7,8 %) avec une différence significative (p<0,001) (Tableau I).

**Tableau I:** Principaux indicateurs par région sanitaire

	Grand Lomé	Maritime	Plateaux	Centrale	Kara	Savanes	Total	p
	N = 1 113	N = 260	N = 237	N = 200	N = 175	N = 115	N = 2 100	
Âge (années)								<0,0011
min-max	15-85	15-83	15-73	15-76	15-74	21-73	15-85	
médiane (Q1-Q3)	45 (37-52)	43 (36-50)	44 (36-51)	42 (33-49)	42 (34-50)	41 (33-49)	44 (36-51)	
Tranches d'âge (années), n (%)								0,0262
moins de 50	747 (67,1)	189 (72,7)	163 (68,8)	153 (76,5)	130 (74,3)	87 (75,7)	1 469 (70,0)	
50 +	366 (32,9)	71 (27,3)	74 (31,2)	47 (23,5)	45 (25,7)	28 (24,3)	631 (30,0)	
Statut matrimonial, n (%)								<0,0012
vit seul	428 (38,5)	110 (42,3)	81 (34,2)	66 (33,0)	55 (31,4)	35 (30,4)	775 (36,9)	
marié(e)	551 (49,5)	124 (47,7)	127 (53,6)	125 (62,5)	102 (58,3)	77 (67,0)	1 106 (52,7)	
non précisé	134 (12,0)	26 (10,0)	29 (12,2)	9 (4,5)	18 (10,3)	3 (2,6)	219 (10,4)	
Stade clinique OMS, n (%)								<0,0012
stade I-II	577 (51,8)	102 (39,2)	133 (56,1)	76 (38,0)	81 (46,3)	90 (78,3)	1 059 (50,4)	
stade III-IV	200 (18,0)	30 (11,5)	40 (16,9)	59 (29,5)	58 (33,1)	7 (6,0)	394 (18,8)	
non précisé	336 (30,2)	128 (49,3)	64 (27,0)	65 (32,5)	36 (20,6)	18 (15,7)	647 (30,8)	
Changement de régime ARV, n (%)								<0,0012
non	795 (71,4)	214 (82,3)	135 (57,0)	147 (73,5)	94 (53,7)	63 (54,8)	1 448 (69,0)	
oui	318 (28,6)	46 (17,7)	102 (43,0)	53 (26,5)	81 (46,3)	52 (45,2)	652 (31,0)	
Type de formation sanitaire, n (%)								<0,0012
privé/confessionnel/associatif	358 (32,2)	1 (0,4)	22 (9,3)	0 (0,0)	0 (0,0)	59 (51,3)	440 (21,0)	
public	755 (67,8)	259 (99,6)	215 (90,7)	200 (100,0)	175 (100,0)	56 (48,7)	1 660 (79,0)	
Sexe, n (%)								0,1782
féminin	758 (68,1)	171 (65,8)	165 (69,6)	147 (73,5)	133 (76,0)	80 (69,6)	1 454 (69,2)	
masculin	355 (31,9)	89 (34,2)	72 (30,4)	53 (26,5)	42 (24,0)	35 (30,4)	646 (30,8)	
Perdus de vue à 90 jours, n (%)								<0,0012
non PDV	880 (79,1)	211 (81,2)	199 (84,0)	139 (69,5)	134 (76,6)	106 (92,2)	1 669 (79,5)	
PDV	233 (20,9)	49 (18,8)	38 (16,0)	61 (30,5)	41 (23,4)	9 (7,8)	431 (20,5)	

1 Kruskal-Wallis rank sum test;2 Pearson's Chi-squared test

#### Mortalité globale des PVVIH

**Figure 2:**
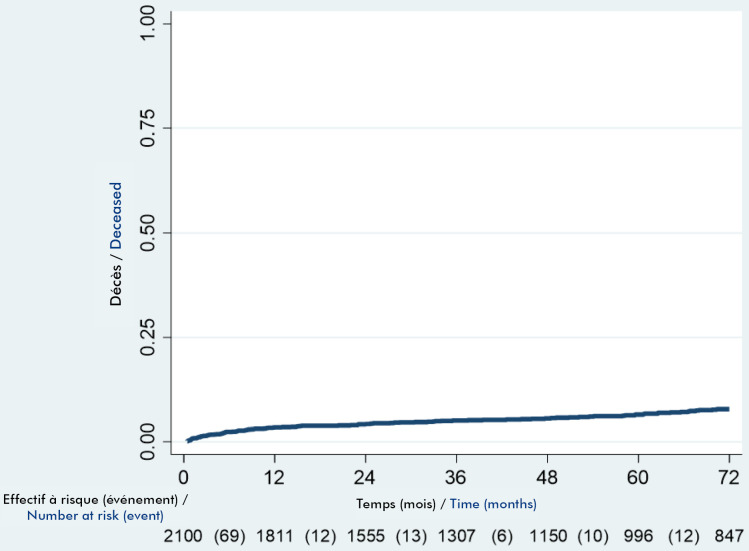
Courbe de survenue des décès chez les adultes infectés par le VIH au Togo

Parmi les patients, 146 adultes étaient décédés, soit une létalité de 6,9 % (IC 5,9-8,1). La probabilité de décès était de 3,5 % à 12 mois, 4,4 % à 24 mois et 5 % à 36 mois (Fig. 2).

#### Devenir des sujets déclarés PDV après 90 jours

Parmi l’échantillon des 158 patients PVVIH tirés au sort, environ 60 % des PDV ont pu être joints au téléphone et continuaient toujours le traitement selon leurs déclarations. Environ 35 % étaient injoignables (ils n'avaient pas laissé de numéro de téléphone, ou en avaient changé) et 5 % étaient décédés selon un membre de la famille.

#### Rétention dans les soins des patients

**Figure 3:**
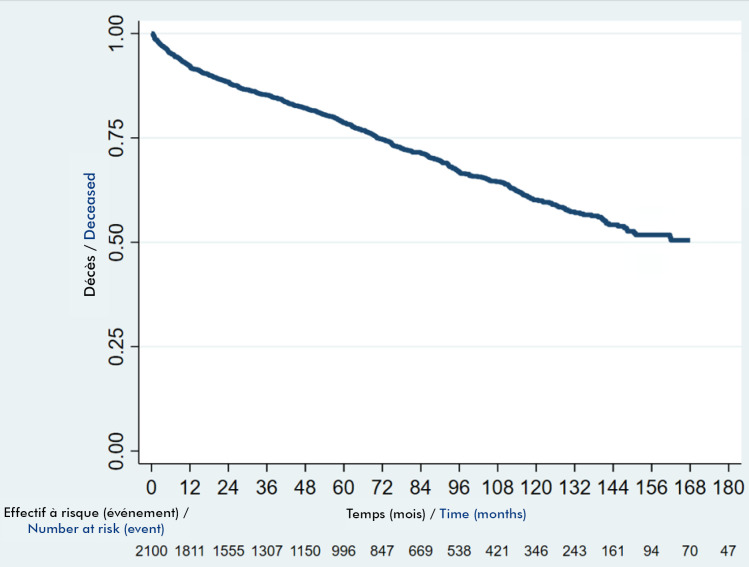
Courbe de rétention chez les adultes infectés par le VIH au Togo

Les patients encore sous TARV étaient 1 523 (72,5 %; IC 70,6-74,4). La probabilité d’être maintenu dans les soins était de 91,6 % à 12 mois et de 87,8 % à 24 mois. A 60 mois, cette probabilité était estimée à 78,7 % (Fig. 3).

**Figure 4:**
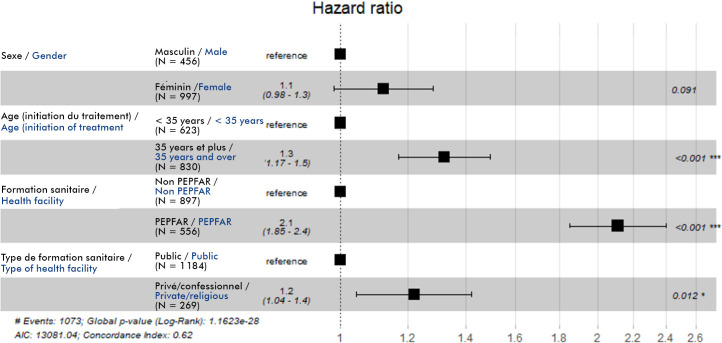
Facteurs associés à la rétention: modèle de régression de Cox (N = 1 453)

*Facteurs associés à la rétention dans les soins* Nous avons inclus 1 453 PVVIH. La rétention dans les soins était plus importante chez les PVVIH âgées de 35 ans et plus lors de l'initiation du traitement *(hazard ratio* (HR)=1,3; p<0,001). De même, elle était plus importante dans les formations sanitaires PEPFAR offrant toute la gamme des activités (soins et traitement, recherche active des patients, présence de médiateurs sociaux) (HR= 2,1; p<0,001) et dans les formations sanitaires de type privé/confessionnel (HR=1,2; p=0,012) (Fig. 4). Ainsi, les facteurs favorablement associés à la rétention dans les soins dans notre étude étaient : être de sexe féminin; être âgé de plus de 35 ans lors de l'initiation du traitement; être suivi dans les formations sanitaires offrant un paquet complet de services (soins, traitement, éducation thérapeutique); bénéficier d'un soutien psycho social.

### Approche qualitative

L'analyse qualitative (interviews et *focus groups)* ont fait ressortir certains facteurs associés aux PDV. Selon les facteurs liés au traitement ARV, les principaux effets indésirables rapportés lors des entretiens sont la somnolence, les vomissements, l'envie de manger à chaque instant, la douleur musculaire, les sensations d'un bruit au niveau du cerveau. Un patient déclarait :

*Quand je prends le produit, je me sens faible, ce qui me pousse à dormir; je transpire beaucoup et à mon réveil tout le lit est trempé. Cette situation a fait qu’à un moment donné j'ai cessé de prendre les ARV.*

Les facteurs socio-économiques, notamment les moyens de déplacement, sont des facteurs associés à la perte de vue dans le programme de soins VIH. Un patient affirmait que :

*On éprouve beaucoup de difficultés pour venir au centre de prise en charge parce que nous manquons des fois de moyens financiers pour payer le transport. Moi par exemple, je suis à plus de 50 km d'ici donc cela fait que parfois j'ai des difficultés à venir pour mes rendez-vous.* Les difficultés financières pour s'alimenter ont été également une des raisons évoquées lors de focus groupe.

Un patient déclarait :

*J'aimerais qu'on mette en exergue dans les conditions socio-économiques, le problème de la famine. Il y a des patients qui vont vous dire que quand ils trouvent à manger, ils prennent bien leur traitement. Mais quand ils n'ont pas trouvé le minimum c'est-à-dire la bouillie même le matin, ils ne vont pas vers leur traitement. Donc dans les conditions socio-économiques, il ne faudrait pas qu'on réduise ça seulement au coup du trajet à effectuer pour venir chercher le produit. Il y a les conditions alimentaires.*

Les voyages apparaissent comme l'une des causes de l'arrêt des soins des PVVIH selon les personnes interviewées au Togo. Quitter son lieu de résidence pour une destination parfois inconnue peut faire manquer le rendez-vous, le patient restant injoignable pendant un long moment. Un patient disait que :

*Moi je dirais que j'ai arrêté mon traitement parce que je n’étais pas au pays. J'avais voyagé au Bénin et mes produits étaient finis et je ne connaissais pas un endroit là-bas pour aller les prendre.*

Par ailleurs, le manque de locaux pour les activités de la prise en charge des PVVIH constitue l'un des problèmes majeurs identifiés par les differents participants à l’étude. De l'avis de l'ensemble des personnes interviewées, l'absence de locaux d'accueil garantissant la confidentialité lors des consultations et de dispensation des médicaments ARV fait que certains patients hésitent à venir pour la prise en charge. Le site de prise en charge étant situé dans un centre public dédié au suivi d'autres maladies, ils craignent d’être exposés devant les autres patients. Un patient déclarait :

*Le lieu de réception est un souci car à la vue de tous, et nous savons tous que la honte nous guette tous. Donc ce qui fait que certains viennent mais n'arrivent pas à entrer dans le centre car trop d'exposition.*

## Discussion

La principale limite de ce travail est due à l'absence de représentativité de l’échantillon qui, certes d'un effectif important, n'est pas aléatoire mais raisonné. Toutefois, la convergence des indicateurs clés suggère une certaine validité des résultats. Cette homogénéité atténue le risque de biais d’échantillonnage et permet d'extrapoler certaines données à une population plus large. La seconde limite est liée au caractère rétrospectif de l’étude avec l'utilisation unique des dossiers et des registres existants non prévus à cet effet. Mais un atout de notre étude est l'effectif important de PVVIH. Une rétention qui est finalement dans la fourchette de notre hypothèse émise (+/- marge d'erreur) pour le calcul de la taille d’échantillon. Les taux de rétention dans les soins des PVVIH sont de 72,5 % (n=1523; IC 95 % = 70,6-74,4). La probabilité d’être maintenu dans les soins à 12 mois est de 91,6 % malgré le contexte de la pandémie de Covid-19. Ces taux sont superposables à ceux obtenus entre 2015 et 2017 qui étaient de 91 % [14]. Plusieurs pays ont été impactés négativement par les problèmes connexes liés à la pandémie de Covid-19 (couvre-feu, limitation des déplacements, peur d'une contamination, problèmes d'approvisionnement en intrants médicaux) avec un effet notable sur l'offre des soins aux PVVIH [1, 4, 6]. Cela n'a pas été le cas pour le Togo. Ceci pourrait être expliqué en partie par l'action des agents de santé communautaires dans la distribution des médicaments ARV au cours de cette pandémie.

Dans notre série, 72,5 % de nos patients continuaient de recevoir le TARV et la probabilité d’être maintenu sous TARV à 12 mois était de 91,6 %, à 24 mois de 87,8 % et à 60 mois de 78,7 %. Ces taux assez faibles justifient la mise en œuvre de mesures dynamiques et innovantes pour améliorer la rétention dans les soins des PVVIH.

Les facteurs favorables associés à la rétention dans les soins ont été documentés dans d'autres pays en Afrique [5, 9, 16] et montrent l'importance de la disponibilité et de la qualité des services pour tous les malades. Le taux de rétention dans les soins était meilleur chez les PVVIH (hommes et femmes) âgées. Cela montre la nécessité de renforcer les programmes d’éducation thérapeutique en particulier chez les plus jeunes. Les problèmes de stigmatisation, les difficultés financières et géographiques pour avoir accès aux soins constituent des facteurs défavorables de rétention dans les soins des PVVIH, d'où l'importance de la décentralisation des services de PEC dans le cadre de la couverture sanitaire universelle aux soins. L'analyse qualitative a aussi relevé un certain nombre d'aspects expliquant la survenue des PDV. Afin d'améliorer la qualité de la PEC des PVVIH dans le pays, il faut mettre en place de nouvelles approches dans les centres de santé. Des interventions (impliquant toutes les parties prenantes au niveau national, communautaire et des établissements de santé) doivent être ciblées pour retrouver les personnes ne revenant pas dans les centres [10]. Au niveau des formations sanitaires, les ARV doivent être disponibles pour 3 à 6 mois de traitement afin de pallier le manque de moyens de déplacement des patients. Par ailleurs, par peur d’être reconnus et stigmatisés, certains patients peuvent être amenés à parcourir de grandes distances pour accéder à un centre de prise en charge éloigné de chez eux, ce qui grève leur faible budget. Il faut promouvoir davantage des interventions contre la stigmatisation au sein de la communauté et encourager les patients à commencer un TARV dans des sites situés à une distance proche de leur domicile [10]. Cela résoudrait en partie les facteurs socio-économiques relevés.

Concernant les effets indésirables des ARV, les autorités sanitaires doivent rendre disponibles les médicaments les mieux tolérés pour susciter l'adhésion des patients et éviter ainsi des interruptions de traitement. Nous pensons que l'utilisation du régime antirétroviral standardisé de première intention comprenant tenofovir + lamivudine + dolutégravir [14] répond à cet objectif.

Les réseaux de PPVIH répondraient aux besoins sociaux individuels en faisant le partage des vécus pour se soutenir et s'encourager mutuellement. Les groupes de soutien renforceraient ainsi le sentiment d'appartenance et le suivi régulier.

De façon pratique, nous pensons que les autorités sanitaires doivent continuer à collaborer avec les organisations non gouvernementales, les communautés locales et les organisations internationales pour renforcer l'encadrement des PVVIH. Le personnel de santé devrait réserver un accueil personnalisé aux PVVIH et établir une communication empathique avec eux. Il devrait contacter les patients avant chaque rendez-vous pour réduire l'absentéisme. Les PVVIH elles-mêmes doivent noter leur rendez-vous et être acteur de leur santé.

Seule l'approche coordonnée et collaborative des différentes parties prenantes pourrait assurer le succès du suivi et de rétention dans les soins.

## Conclusion

Les résultats de notre étude au sortir de la pandémie de la Covid-19 ont permis de documenter les taux de PDV et les facteurs associés à la rétention des PVVIH dans les soins au Togo. Les programmes d’éducation thérapeutique, en particulier chez les jeunes, devraient être renforcés. La recherche active des PDV s'avère efficace.

Les programmes de santé intégrés avec la PEC multidisciplinaire sont indispensables. Les leçons apprises au cours de cette pandémie et les données factuelles issues de cette évaluation ont permis à toutes les parties prenantes de redynamiser les interventions. Ainsi, pour améliorer la qualité de la PEC des PVVIH dans le pays, il a été mis en place des nouvelles approches dans les centres de santé : l'harmonisation des procédures de recherche active des PVVIH avec l'aide des médiateurs communautaires; la dispensation des ARV pour 3 ou 6 mois aux PVVIH; le passage à échelle rapide de l'offre de test de la charge virale. Ces données devront être documentées dans d'autres études à venir.

## Remerciements

Les auteurs remercient le ministère de la Santé, de l'hygiène publique et de l'accès universel aux soins, le Conseil national de lutte contre le VIH/ sida (CNLS), le *Family Health international* (FHI 360) et *Y United States Agency for International Development* (USAID) pour avoir initié cette évaluation dans le cadre de la mise en œuvre du plan stratégique national VIH 2020-2025.

## Financement

Cette étude a bénéficié du financement du gouvernement américain à travers le programme PEPFAR mis en œuvre au Togo.

## Contribution des auteurs et autrices

Akakpo AS, Téclessou JN : rédaction du manuscrit et finalisation.

Deku K, Tchupo JP, Wade S, Ekouevi DK, Adam Z’ Dagnra AY : coordination scientifique générale de l’étude, analyse et interprétation des données et préparation du manuscrit final.

Pitché P : responsable de la coordination scientifique globale de l’étude, rédaction du manuscrit et finalisation.

## Conflit d'intérêt

Les auteurs ne déclarent aucun conflit d'intérêt.
