# Supplementary material for: Analysis of retention in care for people living with HIV in Togo: results of a survey conducted in 2021
Source: Med Trop Sante Int. 2025 Mar 13;5(1):mtsi.v5i1.2025.664. [Article in French] doi: 10.48327/mtsi.v5i1.2025.664 (PMC12012725; doi:10.48327/mtsi.v5i1.2025.664)
Supplement: Related texts

# Research article: Analysis of retention in care for people living with HIV in Togo: results of a survey conducted in 2021

## Introduction

The African Region of the World Health Organization (WHO) alone accounts for more than two-thirds of people living with HIV (PLWH), approximately 25.7 million people in 2019 [10]. Antiretroviral therapy (ART) benefits patients by reducing mortality and morbidity [3]. However, PLWH must not drop out of care, i.e. they must not stop treatment or become lost to follow-up (LTF). By becoming LTF, patients directly endanger not only their own health but also that of others.

According to the WHO, retention in HIV care is defined as a patient's regular engagement in medical care after initiation of ART. The patient is neither LTF nor has died between two visits [11]. Retention in ART care is a major challenge for HIV programs.

The number of patients on ART who are LTF varies from country to country. According to a 2010 study by Ekouevi *et al.* in West Africa (Benin, Côte d’Ivoire, Gambia, Mali, Senegal), the likelihood of retention in care was 90% at 3 months, 84% at 6 months, and 76% at 12 months [2]. However, comparisons between studies should be made with caution, as there is no consensus on how to define LTF in PLWH in general. Among adults, the proportion of LTF ranged from 13.9% to 18.3% in Ethiopia [7, 17] and was 21.2% in Nigeria [13]. In Togo, the retention in care of PLWH on ART 12 months after treatment initiation was 91% according to the results of the Early Warning Indicators (EWIs) study conducted in 2017 [14]. Between 2008 and 2011, Saka *et al.* reported a proportion of PLWH of 1.7% before 6 months and 5.6% after 6 months out of a total of 16,617 patients [15]. As part of the accelerated program implemented in the country, a significant increase in the number of PLWH will be observed by 2021 with a therapeutic coverage rate of 75% [14]. In order to ensure the quality of the care program, it was important to document all aspects of the care program (CP), including the issue of retention in care of patients on treatment in health centers. In addition, the two years (2020 and 2021) of the Covid-19 pandemic strained health systems in Africa including Togo, with a negative impact on programs to manage chronic diseases such as HIV.

Therefore, it was important to conduct this study as the end of the Covid pandemic in Togo. The aim was to estimate, on the one hand, the rate of loss to follow-up of PLWH 28 days and 3 months after initiation of ART and, on the other hand, the rate of retention in CP centers at 6, 12 and 24 months of PLWH, as well as the factors associated with it.

## Methods

### Design, study period and selection of study sites

A mixed approach combining quantitative and qualitative methods was used. It was a cross-sectional analysis of a retrospective cohort of records of PLWH aged 15 years and older who had initiated ART. The retrospective study covered a period of 13 months, from 1/12/2021 to 28/01/2022, in the six health regions of Togo. In 2019, there were 670 HIV care centers. The CP centers were included in the study based on a reasoned selection that took into account the budget available for the study, the accessibility of the centers, and the size of the active patient list at each center. Thus, in each health region, four centers were selected based on the active patient list, i.e. two large CP centers (active patient list ≥ median number of PLWH in the region, i.e. ≥ 50) and two small PEC centers (active patient list < median number of PLWH in the region, i.e. < 50). In addition, in each of the three health regions (Grand Lomé, Maritime and Plateaux) where Ending AIDS in West Africa program (EAWA) centers are located, two of these centers were selected in addition to the previous four. A total of 30 centers were included, with 24,196 people registered in the active file (68,164 in the country as a whole) [13]. Centers with less than 30 PLWH were not selected.

### Study population

For the quantitative survey, a total of 2,100 files were included using a sampling approach designed to take into account site and regional weightings and to allow comparison between PEPFAR (President's Emergency Plan for AIDS Relief) and non-PEPFAR sites. PEPFAR provides in Togo the full range of activities: care and treatment of AIDS, active patient search, presence of social workers. The selection of files was proportional to the size of the active queue per region. The distribution of the sample assigned to the regions was proportional to the size of the active queue. At each site, the number of files was selected by simple randomization.

According to the literature, retention in HIV care in Africa generally ranges from 60% to 80%. For a 95% confidence level, an expected retention rate of 70% with a 2% margin of error, and allowing for 10% unusable data, a minimum of 1,831 patients had to be included.

The sample size was calculated using the following formula:

n=[Z^2^*p (1-p)]/Δ^2^
n: minimum sample size to be included,
Z: confidence level (the standard value of the 95% confidence level will be 1.96),
p: expected proportion of the population with the characteristic under study,
Δ: margin of error [11].

For the qualitative survey, in-depth individual interviews and focus groups were conducted with a convenience sample drawn from different groups of people: patients LTF but located, ART providers (doctors, pharmacists, nurses, etc.), and community and social workers at the study sites. A total of three focus group discussions (FGDs) were conducted per region: one FGD with 5 to 10 PLWH LTF and found; one FGD with 5 to 10 people involved in the care of PLWH on ART; one FGD per region with 5 to 10 community workers.

### Definition of concepts

- Retention in care: all PLWH still on ART who have not died or been lost to follow-up (statistically defined as [1-(proportion of LTFs + proportion of deaths)]).
- Gross retention: proportion of people who have not been LTF nor died at the time of the survey.
- Probability of being retained in care at time i: probability that out of 100 people entering care, they are still being followed up at time i.
- Lost to follow-up (LTF): a PLWH who has not been seen at the center for at least 90 days (3 months) from the scheduled date of the last appointment. This definition does not include 28-day LTFs, as this is a specific indicator of the EAWA program and not a national recommendation.
- True LTF: PLWH who are declared LTFs at their first care site, but who are not followed up elsewhere.
- Attrition: includes deaths and LTFs (LTFs declared in the file and patients who do not return to the center within 90 days of the scheduled visit date). The retention variable is defined as the complement to 1 of attrition (1-attrition).
- Treatment interruption (TI): absence of a clinical contact (ART dispensing appointment) for 28 days after the date of the last appointment. Treatment interruption at 28 days is a specific indicator measured in the EAWA project.
- Outcome of subjects declared LTF after 90 days: we conducted a telephone follow-up of a randomly selected sample of 158 PLWH (but proportionate to LTF in the six regions) among the patients declared LTF. The search for LTF is carried out by social mediators, community health workers and existing networks of PLWH. Once the patient has been declared LTF by the site, this approach uses cell phones and home visits to find them or to confirm that they are still alive.

### Data collection

The study was conducted from 12/01/2021 to 01/28/2022 by reviewing the files and medical records of patients on ART. In addition, facilitators contacted LTF patients by telephone and used an interview guide to collect information. Focus groups were organized within the following populations: site managers; recovered LTF; community workers; staff involved in care.

For the cohort study, a data extraction sheet was developed with about thirty variables. An encoding of the clinical data was reported on the extraction sheet, mentioning the missing data that were taken into account in the analysis. The data were then entered into a database developed using EpiData software version 4.1.

For the LTF search, a database analysis, a telephone survey by facilitators, and a review of the PLWH notebooks were conducted to cross-check the data and document the reasons for loss to follow-up.

The qualitative interviews were conducted by a team of psychologists using interview guides developed for this purpose.

### Data analysis

Upon completion of data collection, quantitative data were exported for analysis using R software version 4.0.1. Qualitative variables were described as numbers and proportions. Quantitative variables were described by their mean or median and their dispersion parameters (standard deviation and quartiles). The Kaplan-Meier method was used to estimate the probability of death or LTF or retention in care with its 95% confidence interval (CI).

We used Cox regression models to identify factors associated with retention in care. Retention in care was coded as a binary variable, with a “no” modality if the patient was LTF or deceased, and a “yes” modality otherwise. All patients with complete data were included in the model. Hazard ratios were estimated with their 95% CI. In the univariate analysis, variables with a p-value less than 0.2 were included in a multivariate model. Multivariate analysis was performed using a stepwise top-down approach.

Qualitative data from the recordings of individual and group interviews were transcribed using Word software and analyzed using the method of “thematic analysis” [8].

### Ethical considerations

The study protocol was validated by the Bioethics Committee for Health Research (CBRS) of the Togolese Ministry of Health (Opinion No. 053/2021/CBRS of November 18, 2021). Written informed consent was obtained from each person before enrollment in the study. In addition to the consents of the adolescents under 18 years, oral consent was obtained from their parents or guardians before they are enrolled in the focus groups. An identification code was assigned to each patient prior to data entry. Access to the database was password protected. All investigators signed a confidentiality clause at the time of recruitment. This ensured the anonymity of the patients, and the confidentiality of the data collected. The data collection instruments did not contain any information that could compromise patient anonymity.

## Findings

### Quantitative approach

#### Sociodemographic characteristics

A total of 2,100 HIV-infected patients were included in this study. Overall, the indicators (age, sex, marital status, clinical stage, type of facility, LTF) show common trends, but with notable regional variations (Table I). The median age of patients was 44 years (IQR [36-51]), with a statistically significant difference between sex and between regions (p<0.001). 70% of patients were younger than 50 years. Women were younger than men (42 versus 46 years, p<0.001). Twenty percent of the patients included in the study had been diagnosed more than 10 years previously. Viral load data were missing in 96% of patients, and 31% had switched ART regimens. The median duration of the treatment was 5 years (IQR [2-8]) with no statistical difference by sex (p=0.752). According to estimates based on operational definitions, 20.5% of patients were LTF at the 90-day follow-up visit (n=431) and 25.1% were LTF at the 28-day follow-up visit (n=509) (Fig. 1). There is a strong regional variation: higher rate in the Central region (30.5%) and lower rate in the Savannah region (7.8%) with a significant difference (p<0.001) (Table I).

**Table I:** Main indicators by health region

	Grand Lomé	Maritime	Plateaux	Centrale	Kara	Savanes	Total	p
	N = 1 113	N = 260	N = 237	N = 200	N = 175	N = 115	N = 2 100	
Age (years)								<0,0011
min-max	15-85	15-83	15-73	15-76	15-74	21-73	15-85	
median value (Q1-Q3)	45 (37-52)	43 (36-50)	44 (36-51)	42 (33-49)	42 (34-50)	41 (33-49)	44 (36-51)	
Age groups (years), n (%)								0,0262
less than 50	747 (67,1)	189 (72,7)	163 (68,8)	153 (76,5)	130 (74,3)	87 (75,7)	1 469 (70,0)	
50 and over	366 (32,9)	71 (27,3)	74 (31,2)	47 (23,5)	45 (25,7)	28 (24,3)	631 (30,0)	
Marital status, n (%)								<0,0012
lives alone	428 (38,5)	110 (42,3)	81 (34,2)	66 (33,0)	55 (31,4)	35 (30,4)	775 (36,9)	
married/in a relationship	551 (49,5)	124 (47,7)	127 (53,6)	125 (62,5)	102 (58,3)	77 (67,0)	1 106 (52,7)	
not specified	134 (12,0)	26 (10,0)	29 (12,2)	9 (4,5)	18 (10,3)	3 (2,6)	219 (10,4)	
WHO clinical staging								<0,0012
WHO Stage I-II	577 (51,8)	102 (39,2)	133 (56,1)	76 (38,0)	81 (46,3)	90 (78,3)	1 059 (50,4)	
WHO Stage III-IV	200 (18,0)	30 (11,5)	40 (16,9)	59 (29,5)	58 (33,1)	7 (6,0)	394 (18,8)	
not specified	336 (30,2)	128 (49,3)	64 (27,0)	65 (32,5)	36 (20,6)	18 (15,7)	647 (30,8)	
Change of ARV regimen, n (%)								<0,0012
no	795 (71,4)	214 (82,3)	135 (57,0)	147 (73,5)	94 (53,7)	63 (54,8)	1 448 (69,0)	
yes	318 (28,6)	46 (17,7)	102 (43,0)	53 (26,5)	81 (46,3)	52 (45,2)	652 (31,0)	
Type of health facility, n (%)								<0,0012
private/religious/non-profit	358 (32,2)	1 (0,4)	22 (9,3)	0 (0,0)	0 (0,0)	59 (51,3)	440 (21,0)	
public	755 (67,8)	259 (99,6)	215 (90,7)	200 (100,0)	175 (100,0)	56 (48,7)	1 660 (79,0)	
Gender, n (%)								0,1782
female	758 (68,1)	171 (65,8)	165 (69,6)	147 (73,5)	133 (76,0)	80 (69,6)	1 454 (69,2)	
male	355 (31,9)	89 (34,2)	72 (30,4)	53 (26,5)	42 (24,0)	35 (30,4)	646 (30,8)	
Lost to follow-up, n (%)								<0,0012
not LTF	880 (79,1)	211 (81,2)	199 (84,0)	139 (69,5)	134 (76,6)	106 (92,2)	1 669 (79,5)	
LTF	233 (20,9)	49 (18,8)	38 (16,0)	61 (30,5)	41 (23,4)	9 (7,8)	431 (20,5)	

1 Kruskal-Wallis rank sum test;2 Pearson's Chi-squared test

**Figure 1:** Various adult monitoring indicators

#### Overall mortality of PLWH

Among the patients, 146 adults died, resulting in a case fatality rate of 6.9% (CI 5.9-8.1). The probability of death was 3.5% at 12 months, 4.4% at 24 months and 5% at 36 months (Fig. 2).

**Figure 2:** Mortality curve among HIV-infected adults in Togo

#### Outcome of subjects declared LTF after 90 days

Among the sample of 158 randomly selected PLWH, approximately 60% of the LTFs could be contacted by telephone and were still undergoing treatment according to their statements. Approximately 35% could not be reached (they had not left a telephone number, or had changed it) and 5% had died according to a family member.

#### Patients’ retention in care

There were 1,523 patients still on ART (72.5%; CI 70.6-74.4). The probability of being retained in care was 91.6% at 12 months and 87.8% at 24 months. At 60 months, this probability was estimated at 78.7% (Fig. 3).

**Figure 3:** Retention curve for HIV-infected adults in Togo

*Factors associated with retention in care* We included 1,453 PLWH. Retention in care was higher among PLWH aged 35 and over at treatment initiation (hazard ratio (HR)=1.3; p<0.001). It was higher in PEPFAR health facilities offering the full range of activities (care and treatment, active patient tracing, presence of social mediators), (HR=2.1; p<0.001) and in private/denominational health facilities (HR=1.2; p=0.012) (Fig. 4). Thus, the factors favorably associated with retention in care in our study were: being female; being over 35 years of age at the start of treatment; being followed up in health facilities offering a full package of services (care, treatment, therapeutic education); benefiting from psychosocial support.

**Figure 4:** Factors associated with retention: Cox regression model (N = 1 453)

### Qualitative approach

The qualitative analysis (interviews and focus groups) highlighted certain factors associated with LTF.

According to the factors related to ART, the main adverse effects reported during the interviews are drowsiness, vomiting, the constant desire to eat, muscle pain, and sensations of noise in the brain. One patient stated:

*When I take the product, I feel weak, which makes me sleepy; I sweat a lot and when I wake up, the whole bed is soaked. Because of this situation, I stopped taking ARTs at one point.*

Socio-economic factors, particularly transportation, are factors associated with loss of sight in the HIV care program. One patient stated:

*It is very difficult to come to the care center because sometimes we lack the financial means to pay for transportation. For example, I am more than 50 km from here, so sometimes I have difficulty coming for my appointments.*

Financial difficulties in obtaining food were also one of the reasons mentioned in the focus group. One patient stated:

*I would like the problem of hunger to be highlighted in the socio-economic conditions. There are patients who will tell you that when they find food, they take their treatment. But if they have not found the minimum, that is, porridge even in the morning, they do not go to their treatment. So, in the socio-economic conditions, it should not be reduced only to the trip to collect the product. There are the nutritional conditions.*

Travel seems to be one of the reasons why PLWH stop taking their treatment, according to people interviewed in Togo. To leave the place of residence for another, sometimes unknown, can do miss appointments, the patient being not be contacted for a long time. One patient said:

*I would say that I stopped my treatment because I was not at home. I had traveled to Benin and my products had run out and I didn't know where to go to get them.*

In addition, the lack of premises for PLWH care activities is one of the major problems identified by the various participants in the study. According to all interviewees, the lack of facilities that guarantee confidentiality during consultations and the dispensing of ARTs means that some patients are reluctant to come for treatment. Because the treatment site is located in a public center where other diseases are monitored, they fear being exposed in front of other patients. One patient said:

*The reception area is a problem because it is in full view, and we all know that shame awaits us all. So, what happens is that some people come, but they cannot enter the center because it is too exposed*.

## Discussion

The main limitation of this work is the lack of representativeness of the sample, which is large but not random. However, the convergence of the main indicators suggests a certain validity of the results. This homogeneity reduces the risk of sampling bias and allows certain data to be extrapolated to a larger population. The second limitation is related to the retrospective nature of the study, with the exclusive use of existing files and registers not designed for this purpose. However, an advantage of our study is the large number of PLWH. This retention is ultimately within the range of our hypothesis (+/-error margin) for calculating the sample size. The retention rate of PLWH in care is 72.5% (n=1523; 95% CI = 70.6-74.4). The probability of remaining in care at 12 months is 91.6%, despite the context of the Covid-19 pandemic. These rates are comparable to those achieved between 2015 and 2017, which were 91% [14]. Several countries were negatively affected by the problems associated with the Covid-19 pandemic (curfews, travel restrictions, fear of contamination, problems with the supply of medical supplies), with a notable impact on the care of PLWH [1, 4, 6]. This was not the case in Togo. This may be partly explained by the work of community health workers in distributing ARTs during the pandemic.

In our series, 72.5% of our patients remained on ART, and the likelihood of retention was 91.6% at 12 months, 87.8% at 24 months, and 78.7% at 60 months. These relatively low rates justify the implementation of dynamic and innovative measures to improve retention in care for PLWH. The positive factors associated with retention in care have been documented in other countries in Africa [5, 9, 16] and show the importance of availability and quality of services for all patients. Retention in care was better among older PLWH (men and women). This highlights the need to strengthen treatment education programs, especially among younger people. Problems of stigma and financial and geographical difficulties in accessing care are unfavorable factors for retention in care among PLWH, hence the importance of decentralizing PEC services as part of universal health coverage for care.

The qualitative analysis also identified a number of aspects that explain the occurrence of LTFs. To improve the quality of care for PLWH in the country, new approaches need to be implemented in health centers. Interventions (involving all stakeholders at national, community and health facility levels) need to be targeted to find people who do not return to the centers [10]. At the health facility level, ARTs must be available for 3-6 months of treatment to compensate for the lack of transportation for patients. In addition, fear of being identified and stigmatized may force some patients to travel long distances to access a treatment center far from their homes, putting a strain on their already limited budgets. More interventions against stigma in the community need to be promoted and patients need to be encouraged to start ART at sites close to their homes [10]. This would partially address the identified socio-economic factors.

Regarding the adverse effects of ARTs, health authorities need to make the best tolerated drugs available to encourage patient adherence and thus avoid treatment interruptions. We believe that the use of the standardized first-line antiretroviral regimen of tenofovir + lamivudine + dolutegravir [14] meets this objective.

PLWH networks would respond to individual social needs by sharing experiences to support and encourage each other. Support groups would thus strengthen the sense of belonging and regular follow-up.

In practical terms, we believe that health authorities must continue to work with NGOs, local communities and international organizations to strengthen support for PLWH. Health workers should welcome PLWH personally and establish empathetic communication with them. They should contact patients before each appointment to reduce absenteeism. PLWH need to keep track of their appointments and take an active role in their health.

Only a coordinated and collaborative approach by the various stakeholders can ensure the success of follow-up and retention in care.

## Conclusion

The results of our study at the end of the Covid-19 pandemic have made it possible to document the rates of loss to follow-up and the factors associated with retention in care among PLWH in Togo. Therapeutic education programs, especially for young people, should be strengthened. Active search for LTFs is proving effective. Integrated health programs with multidisciplinary case management are essential. The lessons learned during this pandemic and the findings of this evaluation have enabled all stakeholders to reinvigorate interventions. For example, to improve the quality of care for PLWH in the country, new approaches have been implemented in health centers: harmonization of procedures for active search for PLWH with the help of community mediators; provision of ARTs for 3 or 6 months to PLWH; rapid scale-up of viral load testing. These data need to be documented in other future studies.

## Acknowledgements

The authors would like to thank the Ministry of Health, Public Hygiene and Universal Access to Healthcare, the National Council for the Fight against HIV/AIDS (CNLS), Family Health International (FHI 360) and the United States Agency for International Development (USAID) for initiating this evaluation as part of the implementation of the National Strategic Plan HIV 2020-2025.

## Funding

This study was funded by the United States Government through the PEPFAR program implemented in Togo.

## Authors’ contributions

Akakpo AS, Téclessou JN: drafting and revising the manuscript.

Deku K, Tchupo JP, Wade S, Ekouevi DK, Adam Z, and Dagnra AY: overall scientific coordination of the study, data analysis and interpretation, and drafting of the final manuscript.

Pitché P: overall scientific coordination of the study, drafting and finalization of the manuscript.

## Conflicts of interest

The authors declare no competing interests.
